# New potential treatments for protection of pancreatic B-cell function in Type 1 diabetes

**DOI:** 10.1111/j.1464-5491.2008.02556.x

**Published:** 2008-11

**Authors:** S Cernea, P Pozzilli

**Affiliations:** Yale University, Department of ImmunobiologyNew Haven, CT, USA; * Department of Endocrinology and Diabetes, University Campus Bio-MedicoRome, Italy; † Institute of Cell and Molecular Science, Barts and The London School of MedicineLondon, UK

**Keywords:** B-cell, immunotherapy, Type 1 diabetes

## Abstract

Type 1 diabetes mellitus results from the progressive and specific autoimmune destruction of insulin-secreting pancreatic B-cells, which develops over a period of years and continues after the initial clinical presentation. The ultimate goal of therapeutic intervention is prevention or reversal of the disease by the arrest of autoimmunity and by preservation/restoration of B-cell mass and function. Recent clinical trials of antigen-specific or non-specific immune therapies have proved that modulation of islet specific autoimmunity in humans and prevention of insulin secretion loss in the short term after the onset of disease is achievable. The identification of suitable candidates for therapy, appropriate dosage and timing, specificity of intervention and the side-effect profile are crucial for the success of any approach. Considering the complexity of the disease, it is likely that a rationally designed approach of combined immune-based therapies that target suppression of B-cell specific autoreactivity and maintenance of immune tolerance, coupled with islet regeneration or replacement of the destroyed B-cell mass, will prove to be most effective in causing remission/reversal of disease in a durable fashion.

## Pancreatic B-cell destruction, residual B-cell function and timing of intervention

During the last decades of the 20th century, there was an increase in the incidence of Type 1 diabetes mellitus (T1D) in most regions of the world. Various environmental factors have been suggested to contribute to this increasing trend (Table 1) [1–4]. Complex interactions between genetic susceptibility and environment, influencing the immunological compartment during the prodromal period, lead in some individuals to overt immunological abnormalities, including occurrence of autoantibodies and islet-reactive T cells [5,6]. Progression of the disease requires activation of the pathogenic T cells and/or a decline in immunoregulation followed by an aggressive attack directed against pancreatic B-cells [6]. Over time, there is a progressive decrease in B-cell mass mirrored by loss of insulin secretion and later by overt hyperglycaemia [7,8]. The Diabetes Control and Complications Trial (DCCT) established support for the relationship between residual B-cell function and glucose control as individuals who had stimulated C-peptide levels > 0.2 pmol/ml had improved responses to treatment and outcomes [9–12]. Thus, preservation of even some endogenous insulin-producing capacity could have a significant impact on the long-term disease outcome by improving glycaemic control. The onset of hyperglycaemia is usually followed by transient partial remission, but the disease continues to develop even after the initial clinical presentation until there is complete insulinopaenia [7,13]. Therefore, early interventions that preclude the onset of the disease (before irreversible destruction of B-cell mass occurs) would be ideal, provided that they are safe and have long-lasting effects.

**Table 1:** The most important environmental factors which possibly contribute to the increased incidence of Type 1 diabetes [1–4]

Environmental factors	Hypothesis
Viral infections (e.g. enteroviruses)	Viral hypothesis
Exposure to certain food constituents (gluten; toxic agents such as nitrite/nitrate)	Dietary hypothesis
Increased hygiene; lack of childhood infections	Hygiene hypothesis
Rapid growth in early childhood	Accelerator hypothesis

The problem with insulin replacement therapy is that it is not curative, as it does not address directly the cause of the disease. The goal of any therapeutic intervention is abrogation of pathogenic reactivity to autoantigens, while preserving full capacity to mount a normal immune response against foreign pathogens, with preservation/restoration of B-cell mass and function. Although a great number of potential therapeutic candidates have been investigated in preclinical models of T1D, many of which showed encouraging results, the successful extrapolation of these findings to humans has proven to be a significantly more difficult task. This is partially because of the huge complexity and inter-individual heterogeneity in the pathogenesis of the human disease, illustrated by the association between younger age at onset and clinical course (shorter duration of symptoms, more severe metabolic decompensation at diagnosis and a more rapid rate of progression) as well as genetic and environmental determinants of the intensity of the B-cell destructive process (greater genetic susceptibility, more intense immune response to B-cell antigens) [14–17]. Therefore, designing therapies that would be effective in all clinical settings is challenging.

Theoretically, therapeutic approaches can be contemplated at different stages of the natural history of the disease. Current dilemmas are optimum time of intervention and proper selection of the candidate population from the perspective of risk/benefit ratio. Low-risk therapies with minimal side effects can be administered ethically before clinical onset (in primary prevention trials), but they require a large number of screened subjects in order to identify a reasonable number of eligible participants, are very expensive and may take a long period of time to document results. In addition, many of the individuals who are genetically predisposed and even have circulating autoantibodies do not develop diabetes or it may occur many years later [7]. Unfortunately, it remains difficult to predict who may benefit from any given intervention, as sufficiently validated and specific surrogate markers of disease prediction or intervention are relatively scarce. It is therefore easiest to perform intervention trials in patients that have been newly diagnosed with T1D as they already carry a straightforward marker of the disease (hyperglycaemia), are certainly highly motivated and the disease course is still amenable to modulation [18]. In addition, these trials require a smaller number of subjects over shorter periods of time to demonstrate the effectiveness of intervention. However, the downside of these interventions is that B-cell damage is already substantial, making potential treatments less effective in arresting the autoimmune process. Thus, the optimal therapeutic approach is the one that ensures a state of equipoise between risk and benefit, balancing the side effects/efficacy ratio.

## Therapeutic strategies for T1D

In this article we will review potential therapeutic strategies for prevention or reversal of T1D in humans with a special emphasis on those that have reached clinical relevance (are either approved for therapeutic use or are being tested in clinical trials of T1D). These approaches broadly address the prevention of (further) B-cell loss by the arrest of the autoimmune process and B-cell protection on one hand and B-cell regeneration and replacement on the other (Table 2). Certainly, the distinction is only scholastic since there is an extensive overlap between these areas and presumably a successful intervention requires a combined approach.

**Table 2:** New therapeutic interventions for Type 1 diabetes in humans

Aim of intervention	Type of intervention		Pros/advantages	Cons/disadvantages
Prevention of B-cell loss	Immune-based therapy	Antigen specific		
		Insulin, HSP60, GAD65 (and their peptides)	Safe Induction imunoregulatory mechanisms Moderate clinical benefit (preservation of C-peptide over a limited time period) in intervention trials	No/partial success in prevention trials so far
		Non-antigen specific		
		Immunosuppresants	Induce depletion/inactivation of pathogenic cells	Limited/no effect; short- and long-term side effects
		Nicotinamide	Safe Protection against B-cell death	No efficacy for diabetes prevention
		Anti-CD3 mAb	Induction of tolerance by apoptosis/ anergy of activated T cells and expansion of Tregs No chronic immunosuppression	Side effects (moderate cytokine release syndrome; transient reactivation of EBV; potential anti-idiotypic antibodies)
			Prevention of loss of insulin production > 1 year in intervention trials	Reoccurrence of B-cell failure
Regeneration/restoration of B-cell mass	Transplantation	Pancreas/islet cells	Feasible Improved glycaemic control Preferable in simultaneous renal graft (when immunosuppression is already necessary)	Modest success of the procedure; continuous immunosuppression is needed Islet isolation procedure—technically challenging; costly; lack of sufficient pancreata (multiple donors per recipient) Whole pancreas—more invasive surgery
	Pharmacologic agents (e.g. GLP-1 receptor agonists, DPP-4 inhibitors)		Approved for Type 2 diabetes therapy Eliminate need for surgical procedures Presumably stimulates B-cell regeneration	Definite effect of B-cell regeneration not yet fully evaluated Long-term safety needs further evaluation
	Stem cells/genetic therapy		Good potential source of surrogate insulin producing cells	Still in preclinical research phase

DPP-4, dipeptidyl peptidase 4; EBV, Epstein–Barr virus; GLP-1, glucagon-like peptide 1; mAb, monoclonal antibody; Tregs, regulatory T cells; HSP, heat shock protein; GAD, glutamic acid decaboxylase.

### Prevention of pancreatic B-cell loss

**Figure 1:**
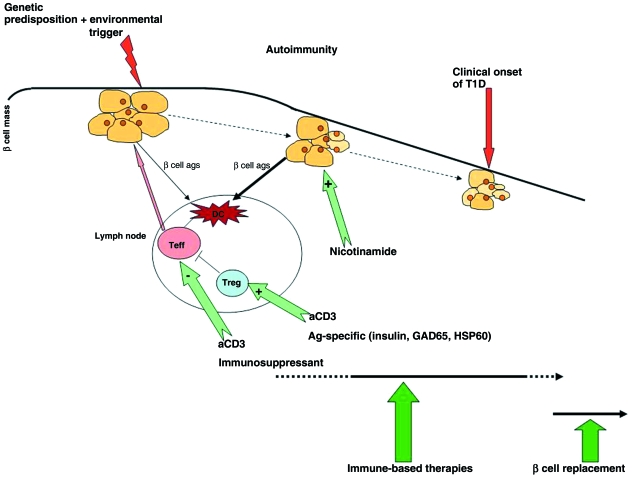
Potential therapeutic strategies for prevention/reversal of Type 1 diabetes. These could be contemplated in different stages during the natural history of diabetes: in individuals with high genetic risk; for prevention of autoimmunity, in antibody-positive individuals; for prevention of disease onset (primary prevention) or after clinical onset; for preservation of residual pancreatic B-cell mass and function (secondary and tertiary prevention). Ideally, immune-based therapies should be administered before onset of Type 1 diabetes (or shortly afterwards), when there is still some residual B-cell mass that could be rescued. Methods of B-cell replacement are used when there is insufficient B-cell mass that could assure sufficient endogenous insulin levels necessary for maintenance of good glycaemic control.

As mentioned, the strategies for prevention of B-cell loss focus on halting the immune response against B-cells in the pancreatic islets and also on protection of B-cells by making them more resistant to the attack. These immunotherapeutic approaches involve both antigen-specific and non-antigen-specific approaches which target various components of the pathological cascade (Fig. 1).

#### Antigen-specific immunotherapy

As T1D results from the failure to maintain tolerance to autoantigens, targeting them through antigen-specific therapies should provide effective means of controlling the autoimmune process by inducing tolerance and also avoid the unwanted side effects associated with non-specific immunosuppression. The rationale behind administration of autoantigen in a tolerogenic regimen is induction of protective immunity by generation of antigen-specific regulatory T cells (Tregs), which act by releasing inhibitory cytokines [e.g. interleukin (IL)-10, IL-4], by induction of anergy/deletion of autoreactive effector T cells and possibly by deviation of their phenotype towards a non-pathogenic one [19–21]. The antigens used as tolerogens have included insulin, glutamic acid decarboxylase (GAD), heat shock protein (HSP) and their constituent peptides. They have been administered by the subcutaneous, oral or nasal routes and have been tested both in prevention and intervention trials.

The initial success of the studies in animal models of diabetes and of a pilot trial in relatives of patients with T1D (which demonstrated that insulin given before disease onset prevents or delays diabetes) led to the initiation of a large-scale clinical trial of an antigen-based therapy for the prevention of diabetes [22–24]. The Diabetes Prevention Trial—Type 1 (DPT-1) recruited high-risk relatives of patients with T1D. Participants were randomly assigned to observation or intervention with oral or parenteral insulin on the basis of their risk of disease [25,26]. Parenteral insulin failed to protect against the disease onset. Overall, oral insulin did not prevent diabetes in all subjects recruited for that arm of the trial; however, a subsequent analysis indicated a delay in diabetes onset in the subgroup of individuals who had high titres of anti-insulin autoantibodies. Possible explanations for the overall study failure are dosage (which were empirically calculated based on animal data and appeared to be 5–8 times lower than the weekly equivalent dose in mice), schedule of insulin administration, changes in the protocol and also timing of the intervention or other poorly defined aspects of the autoimmune response [27–29]. Although the protection was not complete and not all treated subjects benefited, this initial partial success raised hopes for future studies meant to enhance the treatment effect. Type 1 Diabetes TrialNet (http://www.diabetestrialnet.org) has already initiated further studies to explore the potential role of oral insulin in delaying or preventing T1D in relatives whose risk for diabetes is similar to those in the DPT-1 subgroup. An initial report in subjects at risk for T1D found that intranasal delivery of insulin is safe and induced immune changes consistent with mucosal tolerance to insulin [30]. Additional trials are currently ongoing [such as the Finnish Diabetes Prediction and Prevention Project (DIPP)] or planned [Primary Oral/intranasal INsulin Trial (Pre-POINT)] and aim to determine the efficacy of nasal insulin as well as the optimum insulin dose [31,32].

Other studies have used short-term antigen-specific interventions designed to alter the immune response in a manner that might result in sustained beneficial effects. An immunomodulatory humanized peptide from HSP60 (p277 peptide) modified to increase its stability *in vivo* (DiaPep277®), has provided evidence suggestive of better preservation of C-peptide in recent-onset T1D [33]. At the end of the 10 months of follow-up, the intervention group had improved mean C-peptide levels and required significantly less exogenous insulin to obtain the same level of glycated haemoglobin (HbA_1c_) as the control group. Interestingly, drug treatment affected the phenotype of the response to DiaPep277® in the immunized subjects, with enhancement of Th2 type cytokine production. Nevertheless, C-peptide levels decreased later in the treated group, although the change was less pronounced than in the placebo group. A similar study carried out in children with T1D had no beneficial effect in preserving B-cell function or improving glycaemic control [34–36]. Likewise, the report of a clinical trial in latent autoimmune diabetes in adults (LADA) patients using an alum-formulated recombinant human GAD65 showed promise, as it increased C-peptide levels and the purported Tregs subset in peripheral blood. The efficacy of this compound is being examined further in TrialNet intervention trials [37].

#### Non-antigen-specific immunotherapy

Because T1D is a heterogenous disease, and as the initial antigenic repertoire as primary target of the immune attack is still not very well defined, considerable efforts have been devoted to non-antigenic immune interventions. Initial trials used broad-spectrum immunosuppressive agents that would deplete or inactivate pathogenic T cells. Cyclosporin, azathioprine, prednisone and anti-thymocytic globulin led to a decrease in insulin requirements and enhanced endogenous B-cell function in patients with recent onset of T1D, but the magnitude and duration of benefits were limited and concern over short- and long-term toxicity limited their use [38–43].

A large multicentre trial carried out in Europe, the European Nicotinamide Diabetes Intervention Trial (ENDIT), employing a similar screening strategy to DPT-1, randomized subjects at risk to observation or treatment with nicotinamide [44]. Nicotinamide had been shown to modify the course of diabetes in preclinical models, presumably by direct reduction of cytokine-mediated B-cell damage [45]. As in DPT-1, the screening approach was very successful in identifying individuals at risk, but nicotinamide was not effective in prevention of diabetes in this cohort.

Noteworthy and promising outcomes have been seen in two studies using two different humanized anti-CD3 monoclonal antibodies (anti-CD3) in new-onset T1D. The two anti-CD3 molecules were modified in an attempt to decrease toxicity, which was seen mainly in the form of cytokine release syndrome [46,47]. Although the exact mechanisms responsible for the actions of the anti-CD3 are still not fully elucidated, several possibilities exist: induction of antigenic modulation, anergy, induction of apoptosis in activated cells and immune tolerance through adaptive Tregs [48,49]. Both phase II trials using the two different anti-CD3 preparations have reported preservation of B-cell function, with maintenance of higher endogenous insulin secretion assessed by C-peptide response and concomitant reduction in HbA_1c_ and insulin usage in the treated group over at least a 1-year period of time [50–52]. In both trials, adverse events associated with treatment included those of cytokine release (fever, headache, gastrointestinal symptoms, rash) that were self-limiting or responsive to symptomatic therapy. In addition, transient reactivation of the Epstein–Barr virus (EBV) with signs and symptoms of mononucleosis and occurrence of anti-idiotype antibodies have been reported [50–52]. It should be noted that, in spite of the overall initial efficacy in prevention of disease progression, the effects of the therapy varied between individuals (with some patients failing to recover endogenous C-peptide secretion from the beginning of the treatment) and eventually the decline of insulin production was inevitable.

At present, additional studies are being conducted with various other non-antigen-specific immunosuppressive/immunomodulatory agents and are in different enrolment phases [e.g. mycophenolate mofetil ± anti-IL-2 receptor monoclonal antibodies (daclizumab), anti-CD20 monoclonal antibodies, thymoglobulin, IL-2 + sirolimus, CTLA4-Ig, IL-1-receptor antagonist][28].

There has been dramatic progress in the field of immune interventions designed to interfere with T1D progression, but unfortunately a single effective immunotherapy has not been identified so far, although some have shown encouraging results. Antigen-specific immunotherapies have proved to be highly specific in modulating B-cell autoimmunity, mainly by inducing regulatory responses, while non-antigen-specific interventions mediate protection by clonal deletion/anergy in activated (pathogenic) T cells, with subsequent differentiation and expansion of Tregs. For both approaches, identification of suitable candidates for therapy, appropriate dosage and timing and specificity of intervention are critical for successful intervention. Nevertheless, considering the complexity of the disease, it is most likely that a combination of immune-based approaches would be most effective in hindering autoimmunity and maintaining immune tolerance by synergistic effects of therapies that may allow a reduction of individual dosages and thus of side effects.

### Restoration of pancreatic B-cell mass

The clinical significance of any interventional therapy for T1D relies on its ability to maintain, or even increase pancreatic B-cell mass and function up to the point of insulin independence. Because at the time of clinical diagnosis a significant proportion of the B-cell mass has been destroyed (although there may be less than has been appreciated because of the dysfunctional islets), restoration of B-cell mass should be attempted along with methods of immune tolerance; this can be accomplished by B-cell regeneration or substitution.

#### B-cell regeneration

It has been suggested that, in the absence of the autoimmune process that causes cell death, B-cell regeneration is achievable mostly by releasing B-cells from glucose toxicity and oxidative stress and by using regeneration-compatible drugs, that is by substitution of steroids and cyclosporine (and probably also sirolimus and tacrolimus) with agents that seem unlikely to interfere with B-cell proliferation (such as anti-CD3, anti-CD20) [53–55]. The DCCT showed that intensive insulin therapy helps sustain endogenous insulin secretion (with stimulated C-peptide levels above the clinically relevant value of 0.2 pmol/ml), suggesting that continuous insulin administration and its glucose-lowering effect is potentially beneficial for B-cell preservation [56]. There are now compelling data to indicate that B-cell mass is dynamic and can expand and contract to meet metabolic demands (e.g. in pregnancy, obesity) but is not clear, however, whether similar islet mass expansion will occur following the arrest of autoimmunity by effective treatment of T1D [57]. In fact, data suggest that following immune therapy there is a slightly enhanced rate of B-cell apoptosis that continues afterwards. This most likely reflects a process already set in motion at diagnosis and which results in progressive decline in B-cell area [58]. This indicates that immune intervention alone is not sufficient for effective B-cell restoration. Stimulation with pharmacological agents or growth factors (such as hepatocyte growth factor or insulin-like growth factors) may be used to achieve B-cell regeneration and many compounds have been investigated for their ability to induce B-cell replication and neogenesis and to limit B-cell apoptosis [59,60]. Therapeutic molecules capable of increasing B-cell mass *in vivo* by systemic use have the advantage of eliminating the need for invasive surgical procedures. In animal models, expansion of islet mass and stimulation of insulin secretion has been shown to occur in response to glucagon-like peptide 1 (GLP-1) and its receptor agonists, exendin 4 [61–63]. In addition, in islet transplant recipients, exendin 4 has stimulated insulin secretion and demonstrated an ability to reduce exogenous insulin requirements. Currently, clinical trials are testing the hypothesis that use of GLP-1 at the time of islet transplantation might be of help in preserving islet mass [64,65]. Exendin 4 and inhibitors of dipeptidyl peptidase 4 (DPP-4 inhibits GLP-1 breakdown) are new drugs already approved for use as adjuvant therapy in Type 2 diabetes. However, their long-term safety, the durability of effects and their ultimate role in the management of Type 2 diabetes all require further assessment [66]. However, the extent of adult B-cell regenerative capacity is not clear and deeper insight into the effect of these molecules (and other compounds with potential benefit for islet mass regeneration) on human B-cell biology is essential before considering their use for therapeutic purposes in T1D.

It is still a matter of debate whether B-cell renewal occurs by self-duplication of pre-existing endocrine cells, transdifferentiation of exocrine cells and/or by proliferation of ductules/adult stem cells with subsequent differentiation into new islet insulin-secreting cells [59,60]. While basic research provides some evidence for both hypotheses, markers for the clear identification of islet cells that generate insulin-secreting progeny remain elusive. It is doubtful that spontaneous transdifferentiation of exocrine cells and/or proliferation of adult stem/progenitor cells occur in humans and, even if they do occur, the extent of these processes are not clinically relevant.

#### B-cell replacement

B-cell replacement by transplantation of whole organ pancreas or islet cells are currently regarded as acceptable therapeutic options for patients with T1D and both have shown benefits in terms of achieving and maintaining good glycaemic control [67]. Whole pancreas transplant can be performed in conjunction with renal transplant in patients with imminent or end-stage renal disease or as a solitary graft, mainly in diabetic patients with frequent severe hypoglycaemia or extreme blood glucose lability, despite optimization of diabetes management [68]. Islet cells transplantation has similar clinical indications and is a more recent, highly specialized procedure involving implantation of islet cell preparations into the recipient's liver sinusoids via the portal vein, under sonographic and fluoroscopic guidance [65,68]. In general, islet cell transplantation is a less invasive procedure but it requires multiple donor organs per recipient in order to transplant an adequate number of islets. This represents a major impediment to widespread application because of the shortage of donor organs [68]. In addition, both transplantation methods require an immunosuppressive regimen in order to avoid graft rejection and this limits the success of the procedures. The Edmonton protocol (currently accepted as important guidelines for islet cell transplantation) uses a cyclosporin- and steroid-free immunosuppressive regimen; however, even in these circumstances, the reported 5-year insulin independent rate is less than 10%[69]. But it should be noted that C-peptide secretion was maintained in 80% of the patients up to 5 years post-islet transplantation and that the hypoglycaemic score, lability index and HbA_1c_ improved significantly in those who retained detectable C-peptide. A trial carried out in several centres of excellence to explore the feasibility of islet transplantation using the Edmonton protocol had indicated that only 44% of the recipients met the primary endpoint, defined as insulin independence with adequate glycaemic control 1 year after the final transplantation and only 31% of patients remained insulin independent at 2 years [70]. Nevertheless, the obvious benefits justify the ongoing efforts to overcome current challenges through research; mainly development of predictive islet potency tests, increase of islet availability, improvement of post-transplant monitoring, enhancement of islet engraftment and, most importantly, further development of less B-cell toxic immunosuppression regimens [65].

Limitation of pancreatic B-cells availability for replacement has turned the attention of researchers towards finding alternative sources of insulin-producing cells. Various strategies are being investigated and genetic engineering of non-pancreatic cells, or transforming stem cells into glucose-sensitive insulin-producing cells, possibly in combination with other treatments, has generated enthusiasm. We have briefly mentioned before some of the sources of islet cell surrogates. There is an increasing body of evidence showing that, in addition to embryonic stem cells, several potential adult cells derived from pancreas, liver, spleen or bone marrow can differentiate into insulin-secreting cells; these cells display some subsets of native B-cell attributes, but are not yet true functional B-cells [59,60,70]. Thus, the main challenge is development of methods for differentiation to completely functional B-cells, which necessitates a deeper understanding of basic stem cell biology and of stem/progenitor cell differentiation pathways. Apart from certain ethical issues, the use of embryonic stem cells raises the risk of tumour formation as a major concern [60]. However, the use of adult cells poses other constraints, mainly that they are usually isolated in small numbers and in general have limited proliferation capacity [60]. In both cases, yields are still less than adequate to satisfy clinical needs. In spite of all difficulties and current limitations, there is evidence that regeneration of B-cells is possible, yet generation of large number of functional glucose-sensing insulin-secreting B-cells suitable for therapies may not be feasible in the immediate future.

## Conclusions

The ultimate goal of any therapeutic intervention is to prevent or reverse T1D by abrogation of pathogenic autoreactivity and by preservation or restoration of the B-cell mass and function to physiologically sufficient levels to maintain stable glucose control. Recent trials using antigen-specific or non-specific interventions have shown some benefit in modulation of the autoimmune process and in preventing the loss of insulin secretion in the short term after diagnosis of T1D. Even with all the enthusiasm and progress in the field, unfortunately a single long-term effective therapy has not been identified. There are still limitations to current strategies, including a lack of suitable markers to predict and monitor the success of interventions, uncertainty about the long-term adverse effects or the duration of treatment effect and the feasibility of restoration of B-cell mass. Moreover, we should remember that T1D is a heterogeneous disease with an age at onset spanning from childhood to adult age, sometimes with clinical features of Type 2 diabetes such as LADA [71]. Therefore, interventions to protect or regenerate B-cell function whilst managing hyperglycaemia may vary depending on the age of onset of the disease and the extent of pancreatic B-cell damage. It is likely that in most cases a rationally designed combination approach with immunotherapeutic methods which target suppression of pathogenic autoreactivity and induction of immunoregulatory pathways coupled with islet regeneration will prove to be most effective. Ideally, the interventions would be specific for T1D, free of adverse effects and effective prior to disease onset, with long-term and clinically meaningful improvements over standard therapies. The success of these approaches will eventually be evaluated by their impact on glycaemic control as this is the definitive determinant of long-term outcome of the disease.

## Competing interests

Nothing to declare.

## Abbreviations

DCCT: Diabetes Control and Complications Trial
DPT-1: Diabetes Prevention Trial—Type 1
GAD: glutamic acid decarboxylase
GLP-1: glucagon-like peptide 1
HbA_1c_: glycated haemoglobin
IL: interleukin
LADA: latent autoimmune diabetes in adults
T1D: Type 1 diabetes mellitus
Tregs: regulatory T cells
